# Functional Characterization of a CRH Missense Mutation Identified in an ADNFLE Family

**DOI:** 10.1371/journal.pone.0061306

**Published:** 2013-04-11

**Authors:** Veronica Sansoni, Matilde Forcella, Alessandra Mozzi, Paola Fusi, Roberto Ambrosini, Luigi Ferini-Strambi, Romina Combi

**Affiliations:** 1 Department of Surgery and Interdisciplinary Medicine, University of Milano-Bicocca, Monza, Italy; 2 Department of Biotechnology and Biosciences, University of Milano-Bicocca, Milano, Italy; 3 Sleep Disorders Centre, Università Vita e Salute San Raffaele, Milano, Italy; University G. D'Annunzio, Italy

## Abstract

Nocturnal frontal lobe epilepsy has been historically considered a channelopathy caused by mutations in subunits of the neuronal nicotinic acetylcholine receptor or in a recently reported potassium channel. However, these mutations account for only a minority of patients, and the existence of at least a new locus for the disease has been demonstrated. In 2005, we detected two nucleotide variations in the promoter of the CRH gene coding for the corticotropin releasing hormone in 7 patients. These variations cosegregated with the disease and were demonstrated to alter the cellular levels of this hormone. Here, we report the identification in an Italian affected family of a novel missense mutation (hpreproCRH p.Pro30Arg) located in the region of the *CRH* coding for the protein pro-sequence. The mutation was detected in heterozygosity in the two affected individuals. *In vitro* assays demonstrated that this mutation results in reduced levels of protein secretion in the short time thus suggesting that mutated people could present an altered capability to respond immediately to stress agents.

## Introduction

Nocturnal frontal lobe epilepsy (NFLE) is an idiopathic partial epilepsy with increased nocturnal instability, first described in 1994 [1]. It is characterized by a wide spectrum of stereotyped motor manifestations of increasing complexity, ranging from brief motor events to major episodes, mostly occurring during non-REM sleep. NFLE, as well as the familial form of the disease named ADNFLE (Autosomal Dominant NFLE)(OMIM #600513;%603204; #605375; #610353), generally develops within the first two decades of life and frequently disappears in adulthood [2]. Until now 12 mutations affecting genes coding for different subunits (α2, α4 and β2) of the neuronal nicotinic acetylcholine receptor (nAChR) have been associated with the pathogenesis of the disease [2]. However, these mutations account for a minority of patients and the existence of additional loci was demonstrated [3]. Very recently, 4 mutations, causing a more severe form of ADNFLE with intellectual disability and psychiatric features, have been detected in the KNCT1 gene (OMIM *608167) encoding a sodium-gated potassium channel subunit [4]. Finally, in a group of NFLE patients we detected two nucleotide variations in the promoter region of the CRH gene (OMIM *122560) co-segregating with the disease and affecting the gene expression, thus suggesting a possible role in the disease pathogenesis [5], [6]. This gene encodes for the Corticotropin-releasing hormone (CRH), a 41-amino acid peptide derived from a 196-amino acid preprohormone and widely distributed throughout the central nervous system [7]–[9]. CRH acts as a neurotransmitter or neuromodulator in extrahypothalamic circuits to integrate a multisystem response to stress that controls numerous behaviours such as locomotor activity, anxiety, food intake, sexual behaviour, sleep, arousal and learning [10]–[12].

To increase our knowledge on the role of CRH in ADNFLE, we performed a mutation screening of the CRH gene in an Italian family showing a typical form of ADNFLE without psychiatric comorbidities (thus excluding an involvement of the KCNT1 gene) and where mutations in the nAChR genes were excluded.

Here, we report, in this family, the identification of a novel missense mutation (hpreproCRH p.Pro30Arg) located in the region of the *CRH* coding for the protein pro-sequence. The mutation was detected in heterozygosity in the two affected individuals. In vitro assays demonstrated that this mutation results in reduced levels of protein secretion in the short time thus suggesting that mutated people could present an altered capability to response immediately to stress agents.

## Materials and Methods

### Sample composition

**Figure 1:**
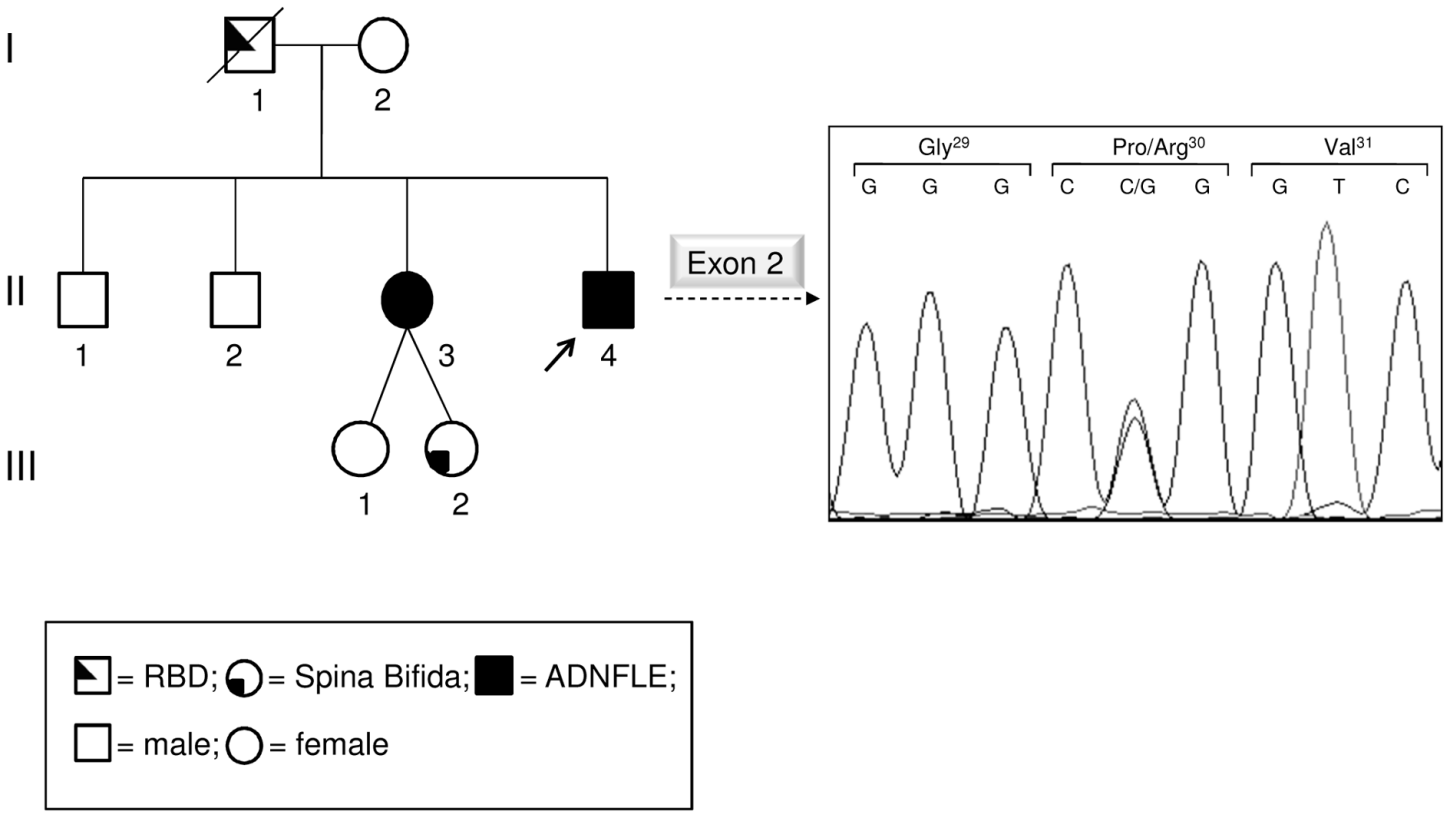
Family pedigree of the proband and electropherograms showing the identified mutation. On the left, the pedigree of the Italian ADNFLE family is shown. The arrow points to the proband. On the right, sequence electropherogram including the identified mutation is shown. The patient is a heterozygote for a missense mutation (p.Pro30Arg). The proband's mother is not a carrier of the mutation and the father genotype is unknown doing to the unavailability of his DNA. RBD: REM sleep behavior disorder.

The sample is composed by an Italian family showing two cases of ADNFLE and a case of spina bifida (Fig. 1). Since the age of 10 years, the family proband had recurrent nocturnal episodes, characterized by a sudden elevation of head and trunk, frequently associated with bimanual and bipedal motor activity. Episodes occurred every night, more frequently in the first third of the night, lasting from 15 to more than 60 sec. Sometimes (4–5 times/month) after the arousal, he would get out of the bed and start wandering around, jumping and making puppet-like movements with his arms. He had no memory of the episodes.

Neurological examination and magnetic resonance imaging (MRI) were normal. Video-polysomnography showed two episodes characterized by an arousal with a sudden elevation of head and trunk and tonic/dystonic posture of the arms: one episode was from stage 2 NREM sleep, and the second one from Slow Wave Sleep. Sleep EEG showed ictal rhythmic slow activity over frontal areas.

The patient has been treated by levetiracetam; with the dose of 1,000 mg at bedtime, the nocturnal seizures were greatly reduced in frequency and complexity.

The sister of the proband experienced similar nocturnal episodes from age 11: until the age of 28, the episodes occurred almost every night (2–3 times/night) and afterwards the frequency was reduced (1–2 episodes/month). She had twin daughters one of which affected by spina bifida.

A group of 100 healthy controls, selected by means of an absent clinical history for the more common diseases and for epilepsy and sleep disorders, was also considered in the study. All individuals were adult and the sex ratio was 1∶1.

The study was approved by an institutional ethical committee (S Raffaele Hospital, Milano, Italy). Written informed consent was obtained from all participants in the study. The study did not involved children/minors.

### Mutation Detection

Polymerase chain reactions (PCRs) were performed directly on genomic DNA. The reaction mixture was prepared according to the protocol of the GoTaq Master Mix (Promega, Madison, WI, USA). PCRs were performed under standard conditions. Primers (Sigma Aldrich St. Louis, MO, USA) were designed from the known genomic sequence of the CRH gene and by means of the Oligo 6.0 software (Molecular Biology Insights Inc, Cascade, CO, USA). Primer sequences are available on request. Sequencing reactions, performed directly on purified PCR products, were performed on both strands by means of the BigDye Terminator Cycle Sequencing kit v1.1 and an automated ABI-3100 DNA sequencer (Applied Biosystems, Foster City, CA, USA). ChromasPro v1.34 (Technelysium Ltd., South Brisbane, Australia) software was used for mutation detection. Sequences were compared with the GenBank sequence NG_016127.1.

### Plasmid constructs and expression vectors

An IMAGE full length human CRH cDNA clone (IRAUup969F078D) cloned into pOTB7 plasmid was purchased by Source Bioscience LifeSciences (Nottingham, UK).

Quick Change II XL Site Directed Mutagenesis Kit (Stratagene, La Jolla, CA, USA) was used to introduce the CRH mutation (c. 89C>G). The cDNA was completely resequenced after mutagenesis to confirm the presence of the desired mutation and to exclude the introduction of other undesired DNA variations.

Plasmids containing the wild-type or the mutated cDNA were purified using the QIAGEN Plasmid Maxiprep kit (QIAGEN, Hilden, Germany) following the suggested protocol and resuspended in water.

Wild-type and mutant cDNAs were then subcloned into the pcDNA3.1 vector, previously cut with EcoRI and XhoI. DNA sequencing confirmed the expected sequence of all constructs.

### Cell cultures and transfection

CRH protein expression and secretion were analysed following transfection of the construct into Neuro2A cells (an established cell line derived from a spontaneous neuroblastoma in an albino strain A mouse), where a correct processing of the proCRH to mature peptide was previously demonstrated [13]. Neuro2A cells of commercial source (Sigma Aldrich) were grown according to standard procedures. Cultures were carried out in DMEM containing 10% fetal bovine serum (FBS), 100 U/ml penicillin, 100 µg/ml streptomycin and 8 mM glutamine. Cell cultures were maintained in 5% CO_2_ humidified atmosphere at 37°C (Thermo Scientific, Waltham, MA, USA).

Transient transfections were performed using the X-tremeGENE 9 DNA Transfection Reagents (Roche, Mannheim, Germany). Cells were plated at a density of 6.5×10^5^ cells per 94 mm plate. Briefly, 5 µg of each expression vectors (pCDNA3X-CRH wt, pCDNA3X-CRHP30R) were transfected using a 3∶1 ratio between X-tremeGENE 9 and DNA. Transfections were performed 24 h after plating and all procedures were according to the manufacturer standard protocol.

### Preparation of cell extracts and cell fractionation

Total lysate extracts were obtained by washing cells in cold phosphate buffered saline (PBS) solution (10 mM K_2_HPO_4_, 150 mM NaCl, pH 7.2) and subsequent lysis with Sample Buffer (50 mM Tris-HCl pH 6.8, 0.4% SDS, 4% Glycerol, 1% β-mercaptoethanol, 0.02% bromophenol blue). The extracts were passed through a syringe needle and then denatured at 100 °C for 5 min.

Cell fractionation were carried out using the Subcellular Protein Fractionation Kit for Cultured Cells (Thermo Scientific) according to the suppliers instructions. The kit allows separation and preparation of cytoplasmic, membrane, nuclear soluble, chromatin-bound and cytoskeletal protein extracts from mammalian cultured cells.

Protein concentration of samples was determined by BCA assay using Pierce BCA Protein assay kit (Thermo Scientific).

Both total lysate and subcellular extracts were used to perform Western blot analysis.

Each experiment has been repeated at least three times.

### SDS-PAGE and Western blot

SDS-PAGE and Western-blot were carried out by standard procedures. PVDF Immobilon™ P (Millipore Billerica, MA, USA) membranes were blocked for 30 min in PBS, containing 5% (w/v) dried milk. Membranes were probed overnight in PBS containing 5% dried milk with anti-CRH rabbit polyclonal antibody (1∶800) (Source Biosciences, Nottingham, UK).

As a control we used anti-α-Tubulin mouse antibody (1∶10000) in TBS-T (50 mM Tris-HCl, 150 mM NaCl, pH 7.5, 0.1% Tween20) containing 5% dried milk and anti-calnexin rabbit antibody (1∶2000) in PBS containing 1% dried milk. Membranes, probed with mouse antibodies, were incubated for 1 h with an anti-mouse horseradish peroxidase-conjugated IgG (1∶10000) (Cell Signalling Technology, Danvers, MA, USA) in PBS, 0.1% (v/v) Tween20 containing 1% (w/v) dried milk, while membranes probed with rabbit antibodies were incubated for 1 h with an anti-rabbit horseradish peroxidase-conjugated IgG (1∶10000) (Cell Signalling Technology) in PBS containing 5% (w/v) dried milk.

Detection of antibody binding was carried out with ECL (Amersham GE Healthcare, Uppsala, Sweden), according to the manufacturer's instructions. Protein levels were quantified by densitometry of scanned not saturated X-ray films using the NIH Image-based software Scion Image (Scion Corporation).

### cDNA synthesis and real-time quantitative PCR

Total RNA was extracted from cultured cells by means of the RNeasy mini kit (QIAGEN) and eluted in water. Synthesis of first-strand cDNA was carried out using Quantitec Reverse Transcription kit (QIAGEN), using 1 µg of total RNA as template. The first-strand cDNA was used as a template for real-time PCR using a human CRH specific primer pair (Fw 5′-GGGAACCTCAACAAGAGCCC-3′ and Rv 5′AACACGCGGAAAAAGTTGGC-3′) and SYBR Green technology (Applied Biosystem). β-actin was used as housekeeping gene (Fw 5′-CGACAGGATGCAGAAGGAG-3′, Rv 5′-ACATCTGCTGGAAGGTGGA-3′). The relative expression levels were calculated with the 2^− [ΔC(t)]^ method.

### ELISA

Indirect enzyme-linked immunosorbent assay (ELISA) was performed to evaluate the presence of mature CRH in the cell culture media. The samples were diluted in 100 mM carbonate/bicarbonate buffer pH 7.4, pipetted into a microtiter plate 50 µL/well and incubated 2 h at room temperature. After then the coating solution was removed and the wells were washed three times with PBS buffer containing 0.05% Tween. The remaining protein-binding sites were blocked with PBS containing 1% BSA and incubated for 2 h at room temperature. After washing twice with PBS buffer, wells were probed overnight in PBS containing 5% (w/v) BSA with anti-CRH rabbit polyclonal antibody (1∶1000) (Source Biosciences).

Then samples were probed with an anti-rabbit horseradish peroxidase-conjugated IgG (1∶10000) (Cell Signalling Technology) in PBS containing 5% (w/v) BSA.

Detection of antibody binding was carried out with TMB (3,3′,5,5′-tetramethylbenzidine) solution for 20 min. The reaction was stopped adding equal volume of 2 M H2SO4. The concentration of CRH was determined by comparing the O.D. of the samples to the standard curve at a wavelength of 450 nm ± 2 nm. Our samples were composed by media collected from cultures of cells transfected with the vector expressing either the wild-type or the mutant CRH precursor.

### Immunofluorescence and confocal analysis

Neuro2A cells were plated onto coverslips (2.5×10^4^ cells/coverslip) and grown for 24 h before transfection. Cells were transfected with X-Treme GENE 9 (Roche) and constructs coding for the wild-type or the mutated form of human CRH using a 3∶1 ratio. 24 and 48 h after transfection, cells were fixed for 20 min in 3% (w/v) paraformaldehyde in PBS and quenched for 30 min with 50 mM NH_4_Cl in PBS. Permeabilization was carried out by incubating the cells in the presence of 0.3% (w/v) saponin in PBS (7 min for 3 times). Cells were then doubly stained with anti-CRH rabbit polyclonal antibody (Source Biosciences, 1∶200). Cells were also incubated with anti-GM130 polyclonal antibody BD Biosciences, 1∶250) for Golgi visualization.

After extensive washes, cells were incubated with Alexa-488 anti-rabbit conjugated antibody (1∶200) and Alexa-555 anti-mouse conjugated antibody (1∶500). All antibodies were from Invitrogen (Carlsbad, CA, USA). Incubations and washes were carried out at room temperature in PBS, 0.3% saponin. Finally, cells were incubated for 15 min with the nuclear marker TO-PRO-3 iodide 1∶300 (Molecular Probes, Invitrogen UK Ltd Paisley, UK). Confocal microscopy was performed using a Leica Mod. TCS-SP2 (Leica Microsystem, Milano, Italy). Image processing was performed with Leica Confocal Software (LCS) and Adobe Photoshop Software (Adobe Systems Incorporated, San Jose, CA, USA). Protein colocalization signals were quantified by densitometry.

### Statistical analysis

Statistical analyses were performed by two-way ANOVAs with genotype (either mutant or wild-type), time (24 h or 48 h) and their interaction as predictors. In no case the removal of the non-significant interaction term altered the significance of main terms. We therefore present the results of the full models only. Robustness of the results to possible deviations from the assumptions of ANOVA test was checked by a randomization procedure (unrestricted resampling of observations for the main terms, unrestricted sampling of residuals for the interaction term, 5000 resamples in both cases; see [14]). Results from the randomization procedure always confirmed those of parametric tests and were therefore not reported for brevity. Post-hoc tests were also performed with the multiple comparison method. Colocalization data were analyzed by a t-test. All the analyses were performed by R 2.15.1 [15].

## Results

### Mutational screening

Sequencing of the coding region, intron-exon boundaries and UTRs of *CRH* revealed that the proband is a heterozygote for a missense mutation (Fig. 1). Nucleotide numbering from here onward is according to cDNA position (GenBank accession number NM_000756.2 starting from the first nucleotide of the ATG start codon); amino acid positions are indicated within the signal peptide and the prosequence.

The mutation consists of a C>G transversion at cDNA position 89 (c.89C>G), which leads to a non-conservative Pro to Arg change at position 30 (p.Pro30Arg according to the Human Genome Variation guidelines) in preproCRH. This mutation was found in the heterozygous state also in the affected proband's sister, while it was absent in the healthy mother (Fig. 1). The father, who was affected from REM sleep behaviour disorder (RBD) and was probably a carrier of the mutation, was unfortunately dead thus it was impossible to verify the presence of the mutation.

The mutation was not present in 100 ancestry-match control samples and it was also not found in public databases. The aminoacid change in CRH was predicted to be pathogenic (PolyPhen2) [16] and affected a highly evolutionary conserved aminoacid.

### Expression and subcellular localization of wild-type and p.Pro30Arg CRH precursor in Neuro2A cells

To evaluate the effect of the identified missense mutation in the production and secretion of CRH, Neuro2A cells, which express only a basal level of endogenous CRH and which are reported to be able to correctly process the prohormone to the mature protein [13], were transiently transfected with plasmid coding either the wild-type or the mutant protein.

Cells lysates were prepared 24 h after transfection and the CRH precursor content was measured by SDS-PAGE Western blot. Results indicated a lower intracellular protein level in cells expressing mutant CRH as compared to those expressing the wild-type form (data not shown).

**Figure 2:**
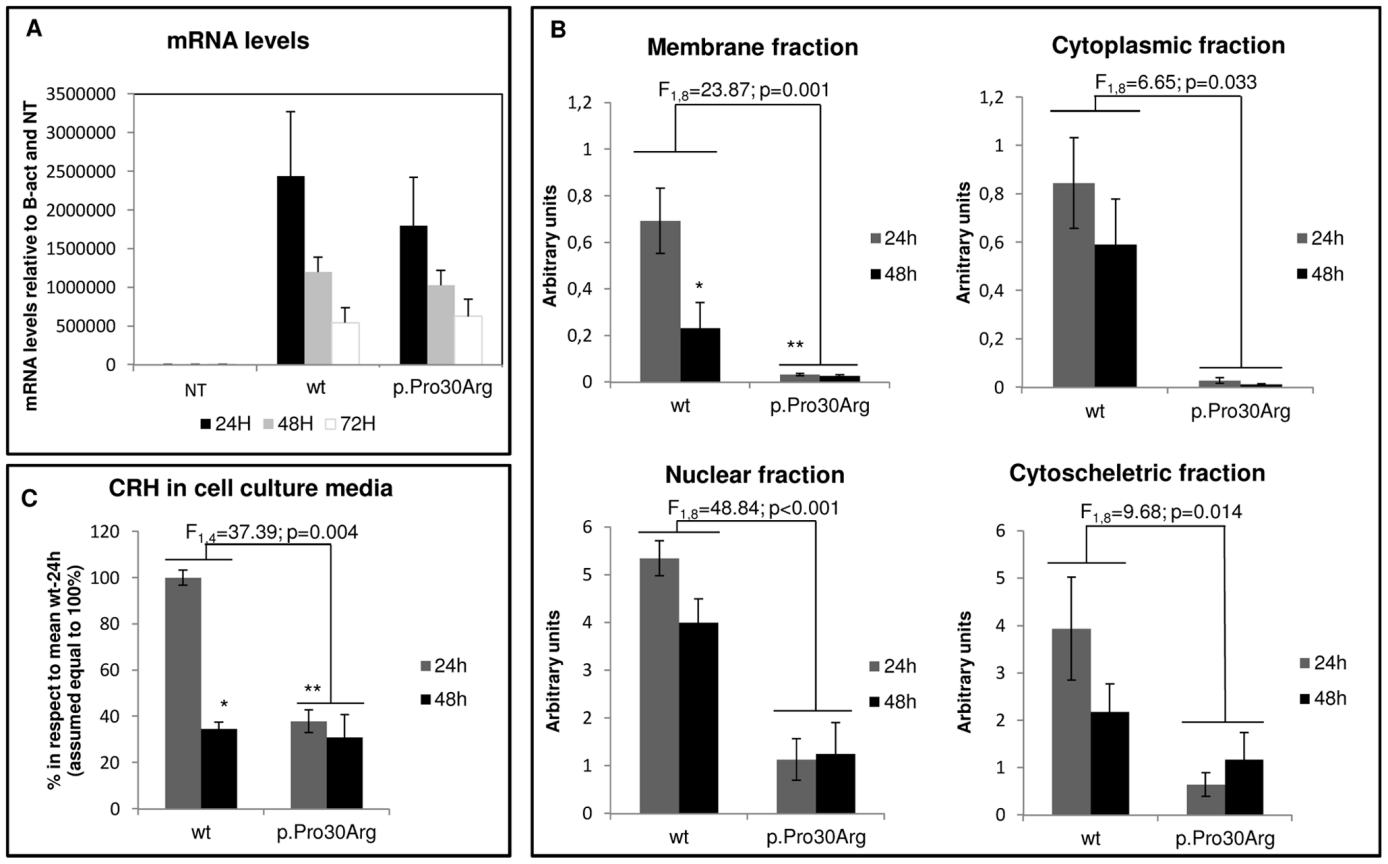
Ability to express CRH in Neuro2A cells transiently transfected with wild-type or mutant preproCRH construct. A) CRH levels of expression detected by realtime quantitative PCR in not transfected (NT) or transfected cells (wt or p.Pro30Arg) at three different times: 24 h, 48 h and 72 h. Each bar represents the mean ± S.E.M. (*n* = 3) of mRNA levels normalized to the basal CRH expression in Neuro2A cells (NT values) and to a housekeeping control gene (b-Actin). * t = −3.676 and p = 0.020 compared with wt at 24 h; ** t = 5.274 and p = 0.002 compared with wt at 24 h. B) Densitometric analysis of CRH immunoreactive proteins in subcellular fractions of the Neuro2A cells. Each bar represents the mean ± S.E.M. (*n* = 3) and protein content is expressed in arbitrary units. C) Levels of secreted CRH protein measured by ELISA. The ability of cells to secrete the CRH hormone was evaluated by measuring the protein level in cultured media of cells transfected either with the wild-type or the mutant construct at 24 h or 48 h after the transfection. Each bar represents the mean ± S.E.M. (*n* = 2) and protein content is expressed as % in respect to the mean value of wt 24 h (assumed equal to 100%).* t = −7.403 and p = 0.005 compared with wt at 24 h; ** t = 7.796 and p = 0.004 compared with wt at 24 h.

No significant differences between the wild-type and mutant cDNA transcription levels were observed in RealTime PCR (Fig. 2A), thus suggesting that the above reported differences were not due to a difference in gene expression.

To test whether the reduction in protein level was generally distributed overall the cell or related to a particular subcellular location, CRH precursor content in extracts from cytoplasmic, membrane, nuclei and cytoskeleton fractions was measured 24 h and 48 h after the transfection.

Statistical analyses showed a significantly lower level of CRH-precursor in all above mentioned fractions of cells expressing mutant CRH in respect to the wild-type, independently of time (F_1,8_ ≥ 6.646, p ≤ 0.033, see Figure 2B for details). Moreover, between 24 h and 48 h, different patterns of variation in the membrane fraction's CRH level were found between the wild-type and the mutant (effect of the genotype by time interaction: F_1,8_ = 6.618, p = 0.033). In particular, post-hoc tests indicated that cells expressing the wild-type CRH precursor had significantly higher protein levels than those expressing the mutant form in the membrane fraction 24 h after transfection (t = 5,274, p = 0.002), while 48 h after transfection no significant differences were detected (t = 1.636, p = 0.360). Protein levels of wild-type CRH precursor decreased significantly between 24 h and 48 h (t = −3.676, p = 0.020) unlike CRH mutant levels (t = −0.038, p>0.999; Figure 2B).

**Figure 3:**
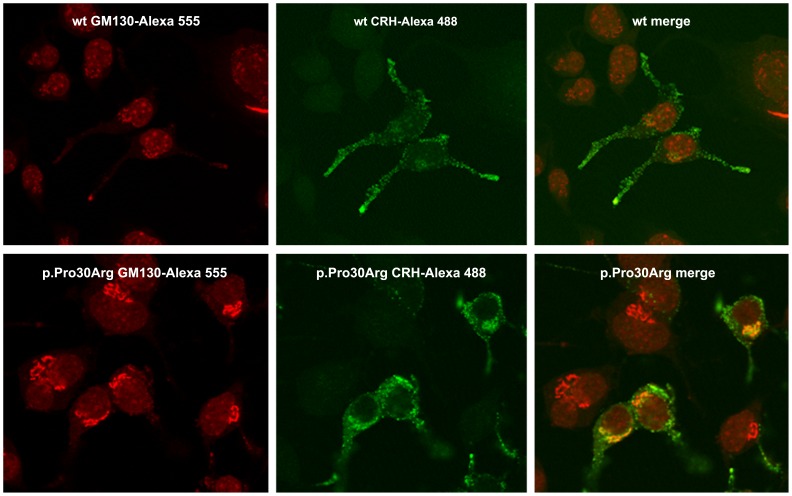
Confocal images of Neuro2A cells transfected with the wild-type or the mutant construct. To investigate intracellular distribution of CRH, cell were fixed in PFA and probed with mouse polyclonal anti-GM130 (red) for Golgi visualization and rabbit polyclonal anti-CRH (green) antibodies.

A difference in CRH intracellular distribution was observed also by immunofluorescence imaging 48 h after transfection. In particular, a statistically significant (t_21_ = 3.406, p = 0.003) higher co-localization with the Golgi apparatus was observed in cells expressing the mutant CRH precursor protein (Fig. 3). The densitometric analysis showed that the average value of the colocalization signal for cells transfected with the mutant plasmid was double than that observed for the wild-type.

### Secretion of CRH in cell culture media

To compare the ability to secrete the hormone of the Neuro2A cells transfected with either wild-type or mutant construct, CRH levels in the culture medium were evaluated 24 h and 48 h after transfection through ELISA. A significant difference in protein levels were observed only at 24 h (F_1,4_ = 37.39; p = 0.004). In particular, at that time protein levels resulted to be significantly lower in media of cells transfected with the mutant construct than with the wild-type (t = 7.796, p = 0.004) (Fig. 2C).

## Discussion

In this article we report a novel mutation (p.Pro30Arg) within the CRH gene cosegregating with sleep disorders (i.e. ADNFLE and RBD) and detected in an Italian family. The mutation was found in heterozygosis in two individuals affected by ADNFLE and it was probably inherited from the dead father (who was reported to suffer of a not precisely defined RBD) being absent in the healthy mother. Unfortunately the DNA of two healthy brothers of the probands was not available. However, even if one of them would be a carrier of the mutation we would have not been able to exclude its involvement in the pathogenesis of the disease due to its well known reduced penetrance. The mutation causes the change of a highly conserved Proline to an Arginine located in the hormone pro-sequence region which is encoded by the exon 2 of the gene. The CRH hormone is in fact firstly produced as a 196-amino acid (aa)-long prepro-CRH, in which the first 26 aa represent the signal peptide, cleaved in the rough endoplasmic reticulum to generate pro-CRH-(27—196) [13]. Endoproteolytic processing of pro-CRH within the trans-Golgi-network and secretory granules generates the mature hormone.

This is the first mutation described in the coding region of the CRH gene and associated to ADNFLE. Up to now two variants in the promoter of this gene were reported in both familial and sporadic cases of NFLE altering the level of the gene expression [2]. The here reported mutation instead, due to its own location, has no effect on the gene transcription levels while it appears to alter the ability of the cell to promptly produce, process and secrete the mature hormone. In particular, a reduced level of CRH immunoreactive peptides was detected both in lysate extracts and in all subcellular fractions derived from cells expressing the mutant cDNA. This could be observed both at 24 h and 48 h after transient transfection. Moreover, our results showed that in the membrane fraction the mutant-protein levels did not vary significantly in the course of time while a significant reduction could be observed in cells expressing the wild-type form. The lack of variation of mutant-protein levels between 24 h and 48 h in this cellular fraction may derive from a functional aspect, arising from a different cellular metabolism, or result from a constitutive low level of the mutant protein expression ab initio.

As far as the reduction in protein levels among the two different genotypes, two possible hypotheses could be put forward: the translation on ribosomes of the mutant mRNA is impaired or the mutant protein is somehow degraded more than the wild-type form. The first hypothesis appears to be less convincing owing to the fact that the mutation is not at the 5′ end of the mRNA and is located far from the translation starting codon. An in silico analysis of the mutation effects performed with Peptide Cutter Tool [17] argued in favor of the second hypothesis owing to the fact that the mutation resulted to introduce a putative cleavage site for three additional proteases (Arg-C Proteinase, Clostripain, Trypsin). Moreover, the half-life of the CRH precursor is very brief thus we could postulate that the mutant protein could not be promptly processed in the rough endoplasmic reticulum and in Golgi apparatus, and this delay could result in a higher level of protein degradation. This delay in processing in presence of the p.Pro30Arg could be related to the identified difference in the membrane fraction's patterns of protein levels: cells expressing the wild-type protein are able to produce and secrete the CRH more quickly than those expressing the mutant form.

The hypothesis of a delay in post-translational protein processing is supported by imaging results showing a higher colocalization between CRH immunoreactive proteins and Golgi apparatus at 48 h after the transfection in cells expressing the mutant than in those expressing the wild-type protein.

Finally, our results demonstrated that levels of secreted CRH were significantly lower for cells expressing mutant CRH at 24 h after the transfection while an apparent recovery could be seen at 48 h when no significant differences were measured. A possible explanation of this recovery could be that, while the wild-type protein is mainly secreted within 24 h, only a reduced amount of the mutant protein (which is less abundant in the cell and “blocked” in the Golgi apparatus) is able to be processed and released rapidly. Instead, the mutation delays this process, thus the majority of the mutant protein is secreted later and it is measured at 48 h in our experiments as the sum of both the delayed mutant protein and the normally produced one. This additive effect masks the intrinsic differences in secretion levels of the two population of cells transfected with different plasmids. It is worthwhile to note that the protein structure of the released mature hormone is the same in the two cases. The mutation, in fact, resides outside the C-terminal domain that produces the mature CRH, thus the incorrect amino acid is intracellularly removed. This location excludes the possibility that the mutation influences CRH properties as a neurotransmitter. Since the mature mutant hormone is identical to the wild-type in structure, it is also very unlikely that its degradation and/or uptake may be affected.

Overall the reported results suggest an impairment in the ability to promptly release the hormone in the presence of the p.Pro30Arg mutation in the pro-sequence. This impairment, which is however partially mitigated in our patients by the fact that the mutation was always found in heterozygosis, could be related to an altered capability of patients to respond quickly to stress agents and this would result in an impaired HPA axis cascade as well as an impairment in the CRH-mediated sleep/arousal cycle regulation. Unfortunately, hormonal dosages in patients positive to the mutation were not feasible because they were already under pharmacological treatments and it is well known that drugs may alter the hormonal levels. A stop of the pharmacological treatment before the hormonal evaluation would have raised ethical questions. Moreover, the peripheral cortisol levels could not reflect those in the CNS.

Although a functional effect of the mutation was demonstrated by our results, a direct role of the p.Pro30Arg in NFLE pathogenesis has still to be proved. This could be done only by the identification of new ADNFLE families with the mutation cosegregating with the disease or by the development and study of specific transgenic mouse models. However, this is the third variant detected in the CRH gene of NFLE/ADNFLE patients causing the production of altered levels of the hormone and this recurrence suggests that individuals with such an altered hormone level could be more prone to develop the disease. In fact, it was previously reported that this hormone promotes wakefulness and impairs sleep in a dose-dependent way [18] and it was also reported as a factor that increases susceptibility to seizures, being however not the direct cause of the seizure onset: altered CRH levels could modify the sigma activity, thus increasing the susceptibility to seizures as well as to abnormal sleep spindles timing. Moreover, the involvement of CRH, which has a much higher proconvulsant effect in young people [19] could be related to the fact that a complete remission of the disease was reported for some patients.

In conclusion, the present paper strengths the importance of a mutation screening of the whole CRH gene in patients affected by NFLE/ADNFLE.
